# Visual imagery of faces and cars in face-selective visual areas

**DOI:** 10.1371/journal.pone.0205041

**Published:** 2018-09-28

**Authors:** Mackenzie A. Sunday, Rankin W. McGugin, Benjamin J. Tamber-Rosenau, Isabel Gauthier

**Affiliations:** 1 Vanderbilt University, Nashville, TN, United States of America; 2 University of Houston, Houston, TX, United States of America; University of Florida, UNITED STATES

## Abstract

Neuroimaging provides a unique tool to investigate otherwise difficult-to-access mental processes like visual imagery. Prior studies support the idea that visual imagery is a top-down reinstatement of visual perception, and it is likely that this extends to object processing. Here we use functional MRI and multi-voxel pattern analysis to ask if mental imagery of cars engages the fusiform face area, similar to what is found during perception. We test only individuals who we assumed could imagine individual car models based on their above-average perceptual abilities with cars. Our results provide evidence that cars are represented differently from common objects in face-selective visual areas, at least in those with above-average car recognition ability. Moreover, pattern classifiers trained on data acquired during imagery can decode the neural response pattern acquired during perception, suggesting that the tested object categories are represented similarly during perception and visual imagery. The results suggest that, even at high-levels of visual processing, visual imagery mirrors perception to some extent, and that face-selective areas may in part support non-face object imagery.

## Introduction

Visual imagery is usually defined as visual perception in the absence of external stimuli. Colloquially, people refer to imagery as “seeing with the mind’s eye.” Visual imagery mirrors perception both in its neural correlates and its behavioral effects [1–3]. Many studies supporting this conclusion use neuroimaging methods, which are uniquely suited to investigate an intrinsic process like visual imagery. Cortical regions associated with particular perceptual acts are also engaged when we imagine the same content. For example, the fusiform face area (FFA) is engaged by both perceived and imagined faces [4–6], the parahippocampal place area by both perceived and imagined places [4,5,7], and motion-sensitive MT/V5 by both perceived and imagined motion [8–10]. Moreover, multi-voxel pattern analysis (MVPA) demonstrated similar representations of perceptual stimuli and their imagined counterparts [7,10–16].

Though most of the functional MRI (fMRI) work on visual imagery has focused on regions in early visual cortex ([14,17–22]; for a review, see [22]), a few studies implicate regions further down the visual processing stream [11,12,23]. In particular, several of these studies have examined regions in and around the fusiform gyrus [4–7,24]. The results support the idea that top-down mechanisms responsible for imagery can engage visual areas at any level of the processing hierarchy depending on the task.

The first study comparing perception and imagery of faces and objects reported considerable overlap between voxels activated during perception and imagery of faces, as well as for perception and imagery of places [4]. A subsequent study found similar overlap between fusiform regions activated during the perception and imagery of faces [6]. However, both of these studies used small samples (eight participants in [4]; nine participants in [6]) and compared face activation with activation for domains specifically chosen because their perception led to different areas being engaged relative to faces (places in [4]; houses and chairs in [6]). Moreover, in both studies, the face-selective regions were not functionally localized which makes it difficult to know if the effects can be assigned to FFA proper. Perhaps the strongest replication of the original study on imagery of faces and scenes by O’Craven and Kanwisher was done by Cichy and coauthors, who compared imagery and perception of bodies, faces, scenes, houses and everyday objects in sixteen participants. They found that extrastriate regions (including the FFA) showed greater activation by their preferred domain during both perception and imagery [5].

Here we aimed to further this line of work by determining whether visual imagery mirrors perception with regard to both face and object representations within face-selective regions. As was done in prior work, we included domains for which we expected distinct activity patterns during perception (faces and everyday objects). Critically, to address our main question, whether imagery of non-face objects at the subordinate-level recruits face-selective areas, we included cars and recruited a sample of participants with above-average car recognition. Based on prior work, we expected cars to be represented in FFA in these participants, since this area is the most robust predictor of behavioral performance in individuation judgments for cars [25–28]. Another improvement we made upon previous neuroimaging studies of face and object imagery is in our definition of face-selective regions. There are two functionally and anatomically distinct face-selective fusiform sub-regions [29–31] that have in the past often been lumped together, although they do not always respond in the same way. In particular, the anterior FFA has been found to show more robust effects of experience for both faces and cars [31–34]. Here, we defined and analyzed these separate regions of interest, to explore how they engage during visual imagery.

We asked if imagery of faces and cars engages face-selective fusiform regions in above-average car recognizers, basing this prediction on evidence that perception of faces and cars engage these regions in comparable populations. In recent work measuring individual differences in the vividness of mental imagery, we found that the vividness of domain-specific imagery (for cars) relates to that of domain-general imagery, but not to perceptual or semantic knowledge ability levels with cars [35]. Therefore, it is unclear whether during car imagery, those with above-average car recognition ability would recruit face-selective areas that are engaged during car perception (where the selectivity has been found to predict car recognition ability), or would recruit only object-selective regions. Note that while studies of perceptual expertise reveal that good car recognizers represent cars in the FFA [25–28], the present work did not include variability in car recognition ability (because car novices are unlikely to be able to imagine different car models with any precision) and as such cannot address this variability. In other words, our use of individuals with above-average car recognition is similar in logic to the use of face experts in most face recognition studies, especially those that rely on famous faces and verify that participants can actually recognize the specific faces used, or to the logic of psychophysical studies that necessitate trained observers. Because our goals differ from studies that characterize expertise effects or study the learning process itself, we intentionally recruited individuals who have good subordinate-level skills for both faces and cars to evaluate the similarity of neural representations during imagery and perception (as opposed to seeking a range of expertise levels).

To address this question, we used MVPA to investigate the information that spatial patterns of neural responses in face-selective regions contain about object categories, when above-average car recognizers imagined faces, cars and a selection of familiar objects. Essentially, MVPA quantifies reproducible spatial patterns of activity that can discriminate between different conditions (see [36] for review). We first used MVPA to determine if representations from these different categories could be distinguished in face-selective areas during perception and during imagery. Critically, MVPA may also be used to demonstrate similarity of representations across conditions by training the MVPA classifier on one discrimination, and testing it on another. We used such an approach here to additionally assess the similarity of imagery and perceptual representations. Specifically, we cross-trained the MVPA classifiers to test if the same patterns of activity that distinguished between categories in imagery also distinguished between categories in perception. Because we expected less overall BOLD activation to be evoked by visual imagery relative to visual perception [4,11,12], analyzing patterns rather than univariate responses provided a more sensitive approach for both of these questions. In addition, MVPA allowed measurement of categorical representational content instead of relative activation amplitude between categories.

MVPA was also useful in overcoming an inherent limitation of our design. Most fMRI work of recognition expertise has been correlational, relating behavioral indices of recognition with the magnitude of region-specific neural activation. However, though individuals with any level of car recognition ability can perceive cars, it is unlikely that individuals with poor car recognition ability can robustly imagine *specific* cars. With MVPA we could investigate the correspondence between perception and imagery in a sample of people capable of imagining cars at a subordinate level. Because we did not compare across levels of car recognition ability, we could not address whether our results would generalize to any individual–indeed, this question may be moot because those with poor car recognition ability could not plausibly perform this task. We could, however, ask whether it is possible to distinguish cars from objects in face-selective areas. To foreshadow our results, we found evidence that imagined cars and objects are represented differently within the face-selective regions in our sample. Moreover, we found evidence that faces, cars and objects are each represented similarly during imagery and perception in these regions, supporting the idea that visual imagery mirrors perception at subordinate levels of object processing.

## Methods

### Participants

A power analysis (using G*power software; [37]) indicated that a sample size of 16 was needed to detect previously reported effect sizes for decoding imagined faces in the FFA with more than 80% power at the .05 alpha level (one sample t-test, Cohen’s d ≈ .78; [5]). We aimed for 20 participants because we do not always find every functional area in all participants. Participants were recruited using flyers posted throughout the Vanderbilt University campus and using ResearchMatch.com. We only recruited males since car interest is predominantly reported by males [38]. Thirty-two men were behaviorally screened and those who showed above-average car recognition ability and visual imagery vividness were invited to participate in the fMRI portion of the study. Of those participants, twenty-one qualified for the fMRI portion and were scanned. One participant did not complete the fMRI portion because of previously undisclosed hearing problems that made it impossible to perceive the auditory stimuli. The remaining 20 participants were all healthy males (mean age = 24.3 years, SD = 6.7, 18 right-handed) who reported normal or corrected-to-normal vision and no hearing loss. Informed written consent was obtained at the beginning of both sessions in accordance with guidelines of the Vanderbilt University Institutional Review Board and Vanderbilt University Medical Center. This study was approved by the Vanderbilt University Institutional Review Board under IRB 050082. All participants received either monetary compensation ($15 for the behavioral screening, $15 for the online tasks, and $45 for the fMRI scan) or course credit.

### MRI Data acquisition

All participants were scanned on a Philips 7-Tesla (7T) Achieva human magnetic resonance scanner with a 32 channel parallel receive array coil (Nova). High-resolution (HR) T1-weighted anatomical volumes were acquired with a 3D TFE (Turbo Field Echo) acquisition sequence with sensitivity encoding (SENSE) (TR = 4.3 ms, TE = 1.90 ms (minimum), flip angle = 7°, sagittal plane acquisition, FOV = 224 mm x 224 mm, matrix size = 224 x 224, slice gap = 0 mm, for an isometric voxel size of 1 mm^3^). During the first participant’s scan,174 slices were acquired, and for all following participants’ scans, 190 slices were acquired. All functional scans were acquired using standard gradient-echo echoplanar T2*-weighted imaging (TR = 2000 ms, TE = 25 ms, flip angle = 65°, axial plane acquisition, FOV = 240 mm x 240 mm, matrix size = 80 x 80, slice gap = 0 mm, for an isometric voxel size of 3 x 3 x 3 mm). By using a comparable functional voxel size (3 mm on a side) to that typical at lower field strengths, we were able to capitalize on the higher field strength (7T) to achieve better signal-to-ratio than would occur at lower field strengths [10]. Following 10 dummy scans, 35 ascending interleaved slices were acquired.

### Stimuli

#### Localizer stimuli

Stimuli used in the localizer runs were greyscale images of 36 unfamiliar faces, 36 common objects, and 83 scrambled images presented centrally against a white background. These stimuli have been used in several previous studies for localizer runs [28,31,39]. None of the common objects or faces used in the localizer runs were also used in the experimental runs.

#### Experimental stimuli

During the experimental runs, participants either perceived or imagined cars, faces or objects. We selected images of 20 faces and 20 objects (none handheld), all relatively easy to imagine based on an online pilot study, and 20 popular car models (all sedans) for the study. We used common objects, similar to previous work [5,11]. For each category, 4 unique stimuli were randomly chosen without replacement for each of the 5 runs, totaling 20 exemplars per category (see Table 1).

**Table 1:** Labels for stimuli from each of the three categories, grouped by which run the stimuli were presented in.

Run	Faces	Objects	Cars
1	Halle Berry	TV	Aston Martin Vanquis
	Bill Clinton	Tent	Volvo V40
	Justin Bieber	Bookshelf	Chevy Impala
	Jennifer Lopez	Lawn Mower	Rolls Royce Ghost
2	Michelle Obama	Park Bench	Honda Accord
	Tom Cruise	Desk	Tesla Model S
	Robin Williams	Chair	Toyota Prius
	Madonna	Water Fountain	Volkswagen Passat
3	George W Bush	Slide	Subaru Legacy
	Taylor Swift	Swing Set	Ford Mustang
	Brad Pitt	Picnic Table	Nissan Altima
	Oprah	Dresser	Pontiac Grand Prix
4	Angelina Jolie	Microwave	Maserati Quattroporte VI
	Leonardo DiCaprio	Pillow	Ford Fusion
	Sandra Bullock	Traffic Light	Volkswagen Beetle
	Jennifer Aniston	Bed	Kia Forte
5	Michael Jackson	Broom	Hyundai Sonata
	Johnny Depp	Boat	Porsche Panamera
	George Clooney	Plane	Toyota Corolla
	Ellen Degeneres	Laptop	Chevrolet Camaro

For the perceptual runs, stimuli were color images of cars, objects, or faces (10 male and 10 female) with the entire background removed using Adobe Photoshop. During the fMRI portion, these stimuli were presented on a black background. For the imagery runs, participants heard recorded auditory labels. For each trial, a .wav file of a male voice saying the label once was played, lasting less than 4 seconds.

### Procedure

#### Behavioral screening

To ensure that participants were able to imagine individual cars, they were screened in a 1-hour preliminary session. During this session, participants completed 4 behavioral tasks (1 measuring visual imagery vividness, 2 measuring performance for cars and other domains, and 1 measure for self-reports of visual expertise). First, participants completed the Vividness of Visual Imagery Questionnaire (VVIQ; [40]) and 2 additional car imagery questions [35]. The VVIQ measures self-reported individual differences in visual imagery and has been used in behavioral (e.g., [41]) and imaging work [18,23]. Next, participants completed a same/different matching task with 3 categories–cars, birds, and houses–followed by the Vanderbilt Expertise Test (VET; [42]) for cars, birds, and butterflies. We chose to use two categories other than cars to better estimate participants’ general object recognition abilities because we were most interested in their car recognition ability controlling for general recognition ability (see [43]).

Participants were included only if they met all of the following cutoffs: 50% accuracy on the VET-car (chance is 33%), a d-prime of at least 1.340 on the car matching tasks, and a score of at least 3 on the VVIQ-general questions. These cutoffs were chosen to be above the corresponding averages from previous larger datasets [42,44–46]. As a group, our sample was chosen to be above average in car recognition ability and also in the vividness of visual imagery. This was important both so participants could perform our task and because there is some evidence that individual differences in vividness can manifest in neural differences [15,18,21,23]. Note that while we recruited participants capable of imagining individual cars from the auditory make and model name cues, our MVPA was done at a categorical level (face vs. car, as done in [4]) not at an individual exemplar level (Ford Fusion vs. Kia Forte, similar to [7]).

To further characterize participants’ recognition abilities and relevant semantic knowledge, independent of the tests used to select participants, they completed the following tasks online a few days before scanning. Participants first completed the Cambridge Face Memory Test long-form (CFMT; [47,48]), followed by the Vanderbilt Face Matching Test (VFMT; [49]), and the Semantic Vanderbilt Expertise Test (SVET; [38]) for cars, birds and dinosaurs. Lastly, participants completed a task to familiarize themselves with the experimental stimuli and their labels. During the first 60 trials, an image was shown with the corresponding label beneath it. Participants studied the image and label and then clicked to advance. There were six 10-trial blocks of each stimulus category (faces, objects, and cars) and the images of a given category were randomized within a block, but presented in the same order to every participant. During the last 60 trials, participants completed a two alternative forced choice to decide which of two labels corresponded to the image shown. Correct responses were randomized with respect to presentation location. This task ensured that participants were familiar with both the images and their labels.

#### Scanning procedure

The MRI portion of the experiment began with a structural run, followed by 2 functional localizer runs and 5 experimental runs. Each participant performed the same tasks with the same stimuli in the same sequence. The localizer runs consisted of 15 16-second blocks with an 8-second block of fixation between each 16-second block, beginning and ending with the task. Each block presented stimuli from either the face, object, or scrambled category and each image was displayed for 900 ms with a 100 ms temporal gap between presentations. Participants completed a 1-back task to detect the one image repeated per block, which they indicated with a button press (right hand index finger). The second localizer run was identical to the first except for the order in which the blocks and stimuli were presented. Each localizer lasted 6 minutes.

**Fig 1:**
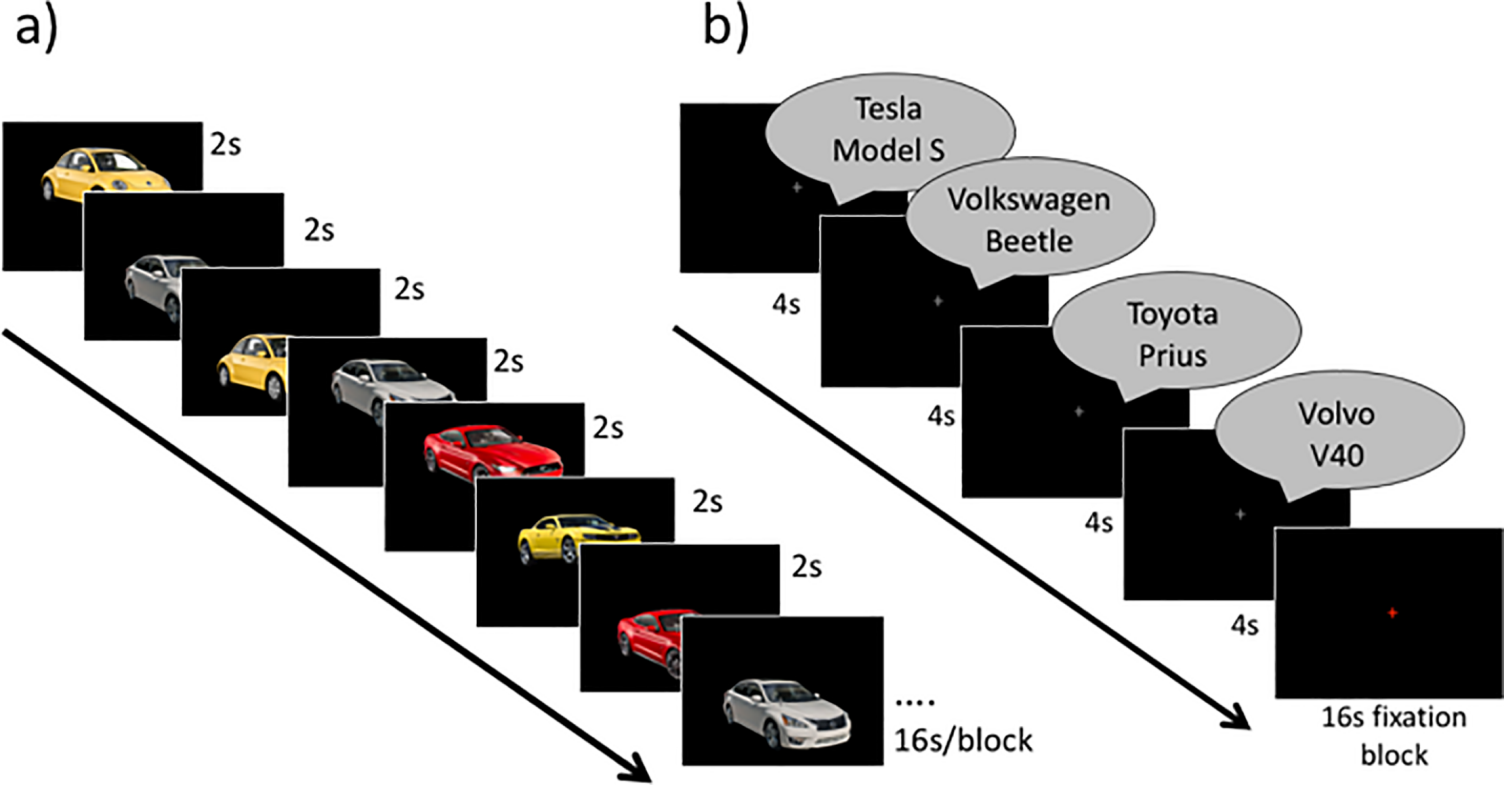
**Schematic of scanning procedure for a sample (a) perception run and (b) imagery run**. In perception runs, participants indicated via key press whether the image was displaced upward or downward.

For both imagery and perception runs, 4 stimuli were chosen from each category (faces, objects, cars) to use in a given run (Table 1). These images (12 total/run) were presented to the participants for review before each run began for approximately 2 minutes. Participants were informed that the images presented on the review slides would be the stimuli used during the following run. Following the two localizers, participants completed an imagery run in which they were instructed to create a mental image corresponding the image label that was aurally presented. The run consisted of 24 blocks of 4 4-second trials each, totaling 96 trials and lasting 6 minutes, 24 seconds. A specific face, object, or car was only presented once per block, while all block and stimulus orders were randomized once and then presented in the same randomized order to all participants. During fixation blocks, participants were instructed to rest, while keeping their eyes open. In imagery runs, a grey fixation cross on a black background changed to red (for one complete 16-second block) to instruct participants to stop imagining and also to encourage participants to keep their eyes open and remain alert despite the minimal visual input. Participants were instructed to keep their eyes open during the entire run so that they would be able to see the fixation cross turn red. Because we wanted participants to focus on creating mental images, the visual imagery itself was the only task participants completed during the imagery runs (Fig 1).

During perception runs, stimuli were presented with a grey fixation cross superimposed over the stimuli, and participants determined if the image was displaced up or down relative to the screen’s center. On each trial, images were displaced either 75 pixels upwards or downwards from the screen’s center, but were always centered horizontally (Fig 1). Participants used a button box to make up or down responses with either their index or middle fingers, respectively. We chose this task to encourage attention to the images while avoiding adding a difficult perceptual/decisional task that was not present in the imagery task. Each perception run consisted of 24 blocks of 8 2-second trials for a total of 192 trials per run. Perception runs, like imagery runs, took 6 minutes, 24 seconds to complete. As with the imagery runs, all blocks and stimuli were randomized. Participants completed 3 imagery runs with 2 perception runs interleaved.

### Data analysis

#### MRI analysis

The HR structural scans were normalized to Talairach space [50]. All functional data were analyzed using Brain Voyager software (www.brainvoyager.com), in-house Matlab scripts, and LibSVM [51]. Functional scans were preprocessed using slice scan time correction (cubic spline, sinc interpolation), 3D motion correction (sinc interpolation), temporal filtering (high-pass filtering with a criterion of 2 cycles per run and a Gaussian filter at 3 seconds), and no spatial smoothing. Across all runs for all participants, we identified all motion-spikes over .5 mm, and any blocks with a spike in motion of this magnitude were removed from all subsequent linear models. Of the 6 participants’ data where spikes in motion were detected, a total of 6 perception run blocks and 7 imagery blocks were removed from analyses. Functional data were registered to the original (non-transformed) structural scan before regions of interest (ROIs) were defined.

Localizer data were submitted to a general linear model (GLM) with regressors for each stimulus category. Face-selective ROIs were defined using the Face>Object contrast from the localizer GLM. We distinguished between two face-selective fusiform areas: a more posterior FFA1 and a more anterior FFA2 [29,30].

**Fig 2:**
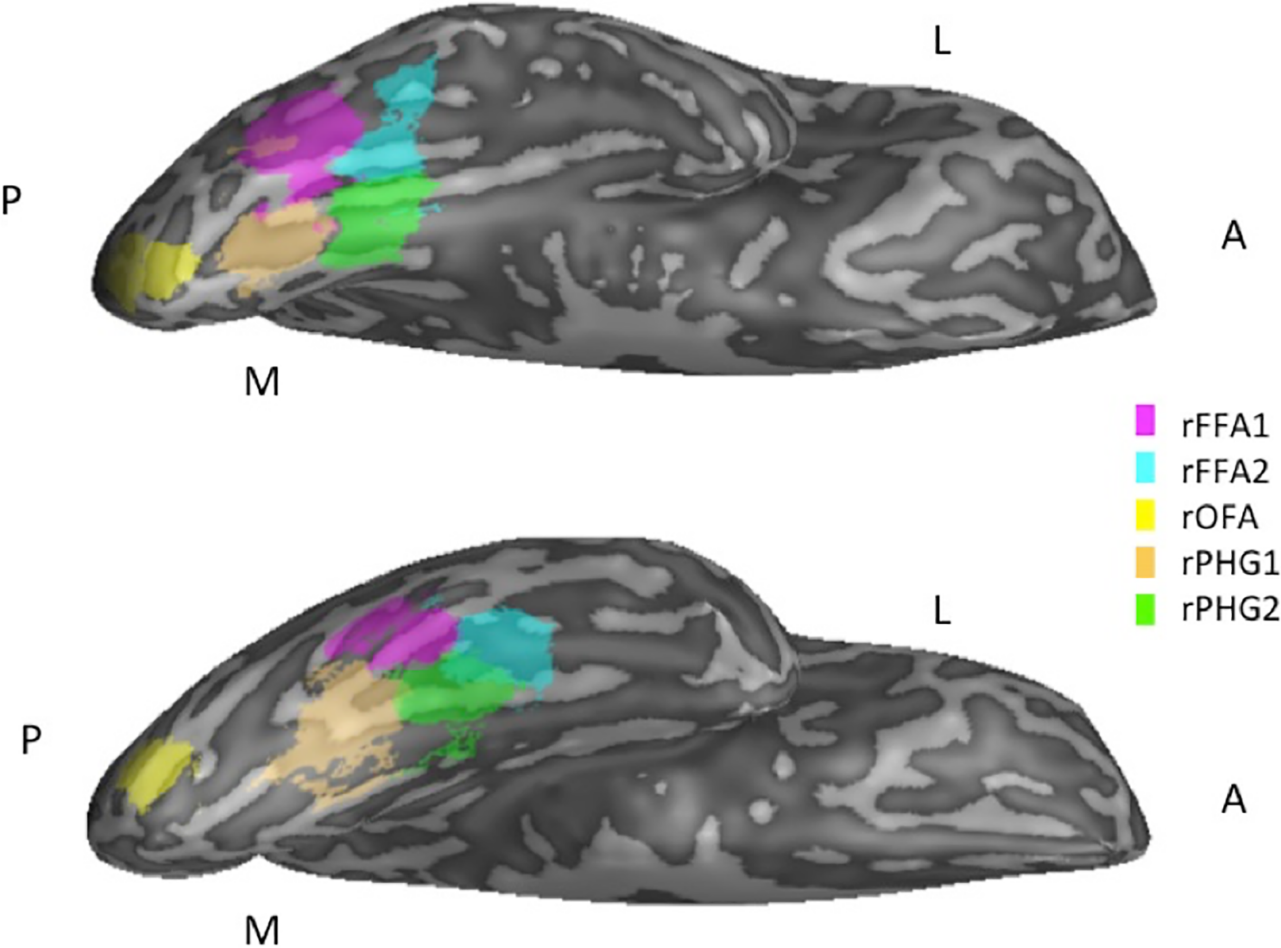
Example 27 functional voxel ROIs shown on 2 inflated right hemispheres of representative participants (anterior and posterior portions of the brain denoted with A and P, medial and lateral with M and L). Note: All ROIs were defined in volumetric space. ROIs transformed to surface space for illustrative purposes only. Any dis-contiguous clusters or ROI overlap that appears in figure did not occur in volumetric space (all ROIs were contiguous and no ROIs contained overlapping voxels).

As is common when defining these functional regions [28,33,34], we could not localize every regions for every participant, and thus only included the ROIs that we could functionally localize. When possible, bilateral FFA1, FFA2, and OFA regions were defined individually for each participant (Fig 2) by finding peaks of significant BOLD responses to faces in the fusiform gyrus. Object-selective regions in the parahippocampal gyrus (PHG) were defined using the Object>Face contrast from the localizer GLM. We defined two object-selective PHG regions (one posterior and one middle, here called PHG1 and PHG2 respectively) bilaterally. Additionally, two lateral occipital object-selective regions were defined using the Object>Scrambled contrast from the localizer GLM. For each ROI, the face- or object-selective peak voxel was identified before the region was grown to 4 functional voxels (108 mm^3^) for univariate analyses or 27 functional voxels (729 mm^3^) for MVPA using in-house Matlab scripts. This script created ROIs by growing regions to include the next-highest contiguous activated voxel until a given size (in this case 4 or 27) was reached. Functional voxels belonging to more than one ROI were removed from both ROIs.

#### Multi-voxel pattern analysis (MVPA)

MVPA was conducted using a linear support-vector machine (SVM). Because we used different subordinate-level exemplars for each run and a blocked design, we trained our classifiers at the category level (e.g. cars vs. objects instead of Kia Forte vs. Honda Accord). We used MVPA to answer two different questions. In an initial series of classification analyses, we asked whether distinct image categories led to different representations in each ROI. Specifically, for each classification, a leave-one-run-out approach was used in which one run was used as test data and the remaining runs were used as training data. Classification rates represent the proportion of times the classifier produced a correct prediction. Because we are interested in object representations during visual imagery, we first focus on our imagery condition. To analyze this imagery condition, the classifier was trained on two runs and tested on one, for the three possible iterations. For the overall classification performance report, an average of these three iterations was calculated. MVPA was done for each participant individually, and results were averaged across participants within equivalent ROIs to form group results. If the classifier is able to distinguish one category from another, this provides evidence that the two categories are represented differently during imagery within that ROI. Most importantly, we were interested in whether imagined cars could be decoded from imagined objects in face-selective regions.

Successful car vs. object decoding during imagery cannot, however, tell us if cars are imagined in a manner similar to the way they are perceived–decoding could be based on any difference in the representations during the imagery conditions. To address this question directly, in a second set of analyses we trained a classifier for each participant using data acquired during the imagery condition and then tested the classifier on data acquired during the perception condition. Prior work with designs that collected equal amounts of imagery and perceptual data obtained better cross-task classification when training on imagery than when training on perception [5]. These authors suggested that all the features of an imagined representation should overlap with those in perceived representations, but that the converse was not true. Based on these prior results, we collected more imagery data and chose to compare perception and imagery by training on imagery and testing on perception. Above-chance performance in such a cross-trained classifier constitutes evidence that 1) the two categories are dissimilarly represented within the given ROI and 2) this dissimilarity is present during both perception and imagery.

#### Univariate analysis

Independent RFX GLMs were fit to the imagery and perception runs with each category (faces, cars, objects and fixation), convolved with the canonical hemodynamic response function, included as a separate regressor. Parameter weights were calculated for each voxel, then averaged across all voxels composing an ROI. We examined all three possible pairwise contrasts of our three stimulus categories: face versus car, face versus object, and car versus object.

## Results

### Behavioral results outside of the scanner

Data are available at https://figshare.com/s/24fc1314649a6e2b2899. Average performance on each behavioral test is reported in Table 2. To compare participants’ car recognition abilities to general object recognition abilities, we averaged the two non-car categories for each task and compared this average to car recognition performance.

**Table 2:** Average accuracy (or d-prime for matching task and rating average for VVIQ and SR) score across participants for each of the behavioral tests, along with standard deviations. Tests used to qualify participants for MRI scan are bolded (N = 20 scanned participants).

Measure	Mean	SD
**VVIQ-General**	**4.09**	**0.39**
VVIQ-Car	4.45	0.47
SR-Car	7.55	1.00
SR-Birds	2.65	1.57
SR-Butterfly	2.00	1.08
**VET-Car**	**0.79**	**0.12**
VET-Bird	0.65	0.12
VET-Butterfly	0.60	0.11
**Matching-Cars**	**2.54**	**0.42**
Matching-Birds	1.56	0.37
Matching-Houses	1.72	0.39
CFMT	0.58	0.16
VFMT	0.59	0.09
SVET-Dino	0.49	0.11
SVET-Bird	0.46	0.08
SVET-Car	0.87	0.14

As expected (given that we screened based on VET-Car and matching task-Car performance), participants performed significantly better with cars than non-car categories (VET: (*t*(19) = 4.98 *p* ≤ .001, d = 2.28; Matching Task: *t*(19) = 8.71; *p* ≤ .001, d = 4.00). While these tasks are not precisely matched in difficulty, in large unscreened samples the VETs have highly similar means (see [42]). Our participants also performed significantly better on the SVET-Car than non-car SVETs (*t*(19) = 12.14; *p* ≤ .001, d = 5.57).

### Behavioral results from the scanner

Performance on behavioral tasks in the scanner indicated that participants were attending to the stimuli. The average performance on the n-back task during the first and second localizer runs (not including scrambled images) was 79% (SD = 19%) and 93% (SD = 8%), respectively. Average performance on the up/down displacement task during the first and second perception runs was 83% (SD = 26%) and 86% (SD = 17%), respectively.

### MRI Results

#### ROI identification

To grow regions suitable for MVPA, we created ROIs composed of 27 functional voxels. Though past work has shown that ROI sizes of around 100 voxels produce optimal MVPA performance [52], some of our functional regions are small and close to one another (e.g., FFA1 and FFA2). Given that typical searchlight MVPA analyses use spheres of approximately 30 voxels [53], we chose to use 27 voxels since this was the largest size at which we could avoid identifying many overlapping voxels across functional region definitions (Table 3). All ROIs were grown to 27 3x3x3 functional, non-overlapping voxels except for the following which were smaller because of dropout due to the ear canal: the lOFAs in two participants (12 functional voxels), the rOFA in one participant (18 functional voxels), and all ROIs in one participant (17 functional voxels). The MVPA results did not qualitatively differ when these smaller ROIs were excluded from analysis. For these reasons, along with the fact that we could not functionally localize each ROI for each participant, we had unequal numbers of ROIs (see Table 3), as is typical in work with these sub-regions (e.g. 33). We used 4-functional-voxel ROIs for our univariate analyses so that we would include only peak activation [34]. However, univariate results with the 27-functional-voxel ROIs produced qualitatively similar results to the smaller ROIs.

**Table 3:** Peak Talairach coordinates of the peak structural voxel for each 27-functional voxel ROIs with 95% confidence intervals reported in parentheses. N column reports the number of participants in whom we localized the respective ROI.

ROI	Peak X	Peak Y	Peak Z	N
rFFA1	37.00 (34.46, 39.55)	-60.73 (-63.88, -57.59)	-17.40 (-19.64, -15.16)	15
rFFA2	36.83 (34.81, 38.86)	-43.94 (-46.77, -41.12)	-20.78 (-22.83, -18.72)	18
rOFA	30.15 (26.41, 33.89)	-77.62 (-80.92, -74.31)	-18.00 (-22.10, -13.90)	13
lFFA1	-39.46 (-41.85, -37.08)	-60.08 (-63.85, -56.31)	-19.38 (-22.48, -16.29)	13
lFFA2	-39.95 (-41.21, -38.69)	-46.00 (-48.22, -43.78)	-20.85 (-23.32, -18.38)	20
lOFA	-37.50 (-39.88, -35.12)	-76.67 (-79.85, -73.48)	-17.92 (-21.26, -14.57)	12
rPHG1	25.70 (23.80, 27.60)	-46.30 (-48.46, -44.14)	-13.05 (-15.40, -10.70)	20
rPHG2	24.60 (22.93, 26.27)	-63.60 (-66.67, -60.53)	-13.80 (-16.11, -11.49)	20
lPHG1	-26.74 (-28.25, -25.22)	-45.58 (-47.95, -43.21)	-16.74 (-19.09, -14.39)	19
lPHG2	-27.21 (-28.82, -25.60)	-62.21 (-64.95, -59.47)	-15.32 (-17.89, -12.74)	19
rLOC	40.85 (38.38, 43.32)	-70.55 (-72.82, -68.28)	-8.85 (-11.46, -6.24)	20
lLOC	-44.50 (-46.52, -42.48)	-69.80 (-70.96, -68.64)	-9.75 (-12.23, -7.27)	20

### Multi-voxel pattern analysis

#### Within-task classification

**Fig 3:**
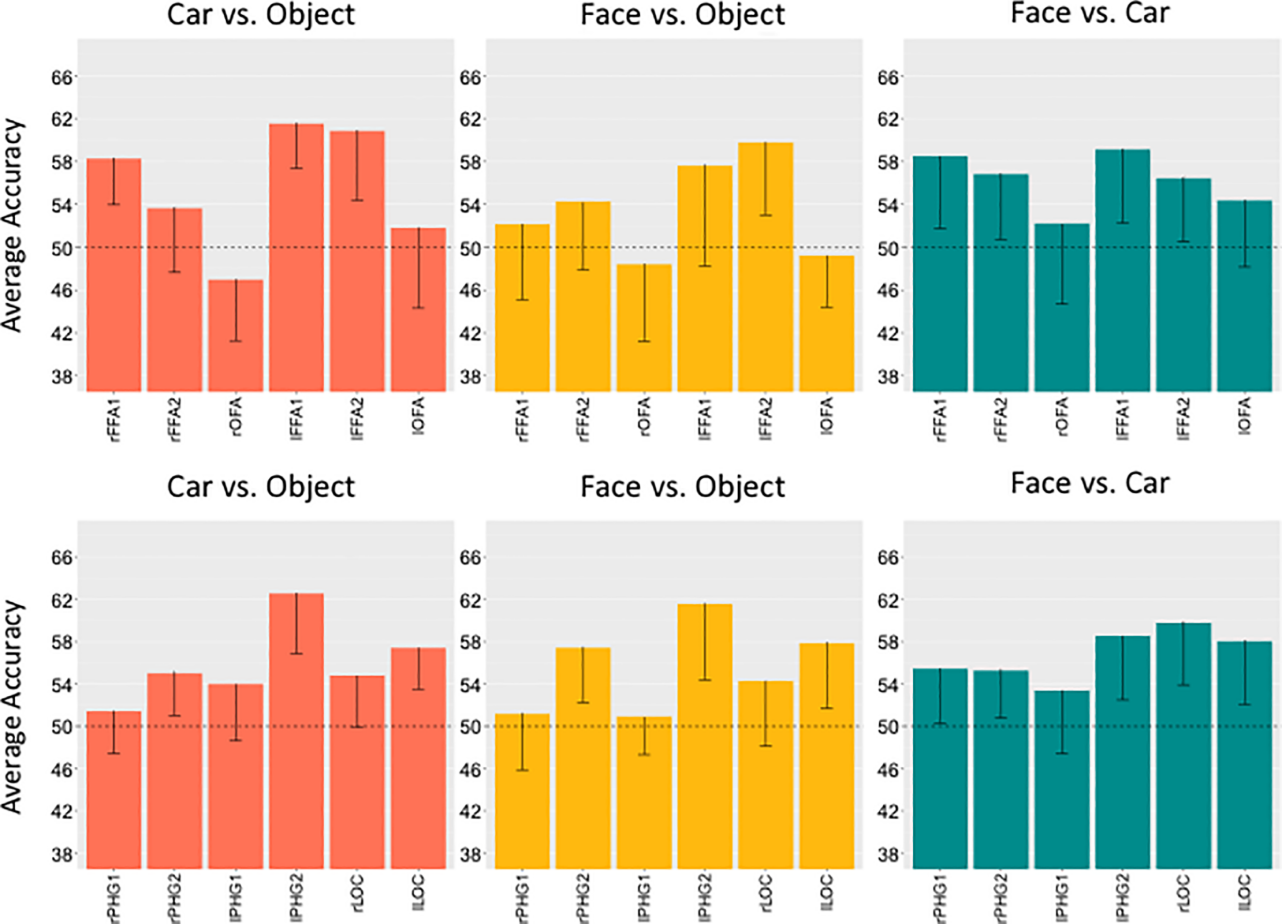
**Average accuracies of the classifier when trained on imagery data and tested on imagery data for object versus car (left) and face versus object (middle) and face versus car (right) two-way classifications in face-selective ROIs (upper row) and object-selective ROIs (lower row)**. Error-bars represent one-tail 95% confidence interval, accuracy of decoding below chance was not theoretically meaningful.

To test if activity patterns for imagined faces, cars and objects were distinguishable, we performed MVPA on the imagery task (i.e., both training and testing data from imagery runs) separately for each ROI and each participant. This addresses whether it is possible to decode cars versus objects in face-selective areas. The classifier achieved above chance performance in both lFFA2 and lFFA1, as well as rFFA1 (Fig 3). Decoding imagined faces from imagined objects was only possible in lFFA2, although the effect in lFFA1 was close in magnitude (note that we had reduced power in lFFA1 because we were only able to localize the ROI in 13 participants, Table 3). Imagined faces and cars could be decoded in all four FFA ROIs. Average MVPA performance (for face vs. car, face vs. object and object vs. car) in face-selective ROIs did not correlate with behaviorally measured car recognition performance across subjects (*r*’s = -.17 –.27, *p*’s > .24). This is unsurprising given that we intentionally selected participants with high car recognition ability.

In object-selective regions, the classifier could decode imagined faces versus imagined objects and imagined cars versus imagined objects in bilateral PHG2 and lLOC (Fig 3). Imagined faces and cars could be decoded in all object-selective ROIs except the lPHG1. Both within-task and across-task analyses of early visual areas provided little evidence that categories could be distinguished in these regions (see supplement for details).

#### Across-task classification

**Fig 4:**
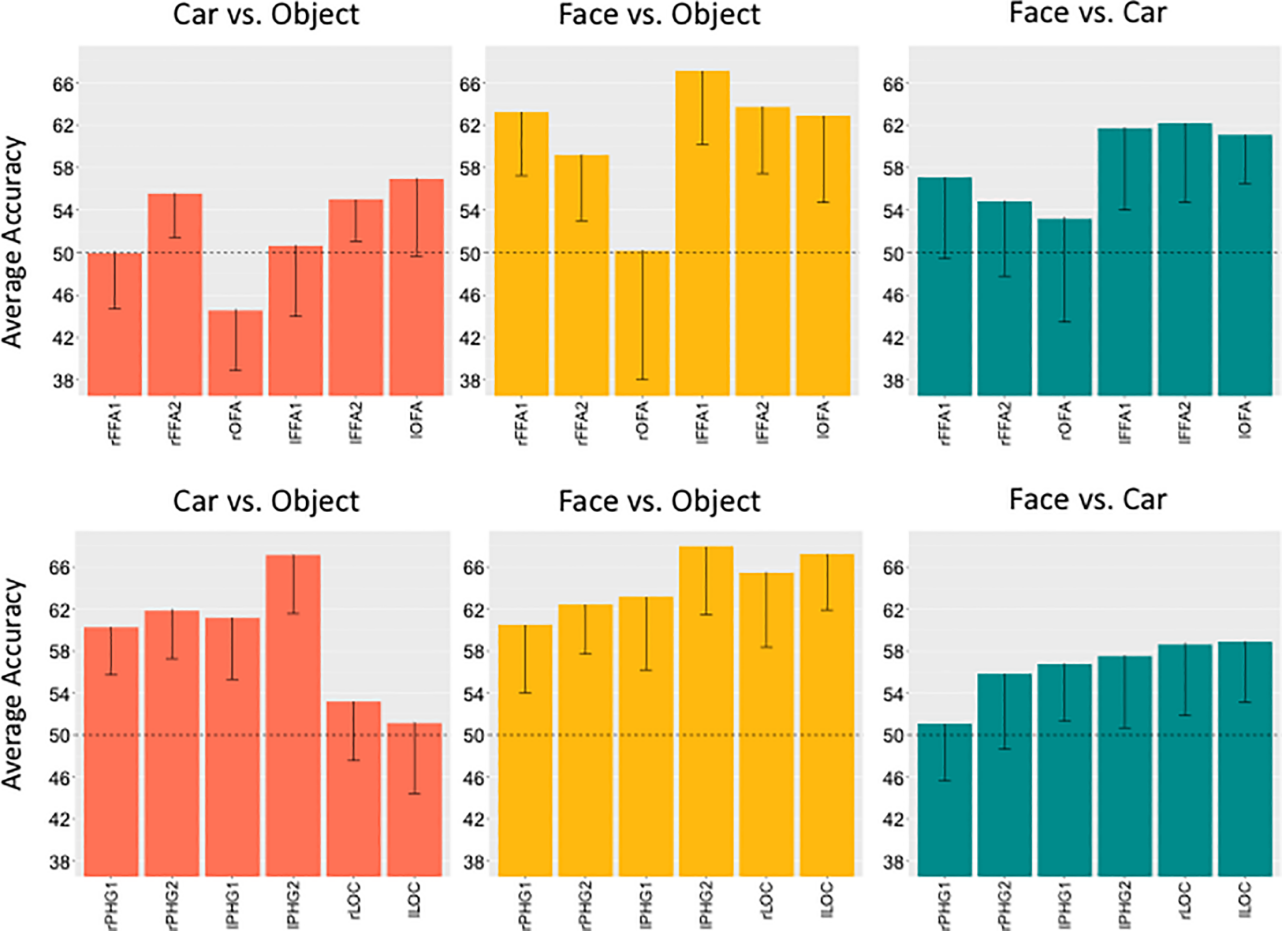
**Average accuracies of the classifier when trained on imagery data and tested on perception data for object versus car (left) and face versus object (middle) and face versus car (right) two-way classifications in face-selective ROIs (upper row) and object-selective ROIs (lower row)**. Error-bars represent one-tail 95% confidence interval, accuracy of decoding below chance was not theoretically meaningful.

To ask whether the categorical differences we observed in imagery reflected the representations evoked by these categories during perception, we trained a classifier with data from the imagery runs and tested its ability to decode the perception runs. Central to our question, we were able to distinguish perceived cars from perceived objects based on imagery information in bilateral FFA2 (Fig 4). Additionally, we could also decode perceived faces from objects based on imagery information in all face-selective ROIs except rOFA (Fig 4). Based on imagery information, perceived faces could only be distinguished from cars in left face-selective ROIs.

In all object-selective regions, perceived faces were decoded from perceived objects based on imagery information, and perceived cars were also distinguished from perceived objects in all but bilateral LOC ROIs (Fig 4). All object-selective ROIs except the rPHG regions could distinguish perceived faces from cars based on the imagery-trained classifier.

### Univariate analyses

The average parameter weights are reported in Table 4. Face and car parameter weights are reported relative to an object baseline. We use objects as a baseline condition so that both low- and high-level visual processing activation would be subtracted out. In contrast with previous work reporting significant activation during face imagery in face-selective fusiform and OFA regions [4–6], we only found significantly greater activation for imagined faces vs. imagined objects in one face-selective region, lFFA2 (Table 4). In addition, when examining the parameter weights for the perception runs (Table 4), we noted that while face stimuli showed the predicted pattern of eliciting higher BOLD response in face-selective areas (FFAs and OFAs) relative to objects and lower responses in object-selective areas (PHGs and LOCs), this was not always true of the car stimuli. Notably, in face-selective areas the response magnitudes for perceived cars relative to perceived objects was not significantly different from zero (Table 4).

**Table 4:** Average GLM parameter weights for both imagery and perception runs. Each weight is the average of the 4 contiguous most face- or object-selective functional voxels (identified via the independent functional localizer) within the ROI. Beta weight is reported along with the *t*-statistic and FDR adjusted *p*-value (*q*-value) of a two-tailed test of the differences being different from 0.

	Perception					Imagery				
	Face-Obj			Car-Obj		Face-Obj			Car-Obj	
	β		*t*	β	*t*	β		*t*	β	*t*
rFFA1	0.84		8.41 (.00)	0.01	0.15 (.90)	0.04		0.27 (.87)	-0.02	-0.23 (.87)
rFFA2	0.68		5.91 (.00)	-0.08	-0.79 (.55)	0.16		2.03 (.12)	0.05	0.65 (.74)
rOFA	0.52		2.70 (.02)	0.22	1.48 (.27)	0.02		0.22 (.87)	0.02	0.16 (.87)
lFFA1	0.80		4.28 (.00)	-0.02	-0.13 (.90)	0.02		0.17 (.87)	-0.03	-0.36 (.87)
lFFA2	0.64		6.44 (.00)	-0.10	-0.91 (.54)	0.32		3.51 (.00)	0.06	0.96 (.74)
lOFA	0.72		2.63 (.02)	0.34	1.48 (.27)	-0.17		-0.78 (.75)	-0.24	-1.54 (.50)
rPHG1	-0.88		-7.08 (.00)	-0.49	-3.18 (.00)	-0.03		-0.45 (.87)	-0.19	-2.54 (.10)
rPHG2	-0.93		-10.52.00)	-0.65	-4.58 (.00)	-0.22		-3.17 (.03)	-0.05	-0.73 (.74)
lPHG1	-0.87		-8.31 (.00)	-0.56	-4.70 (.00)	-0.23		-3.16 (.03)	-0.19	-3.03 (.10)
lPHG2	-0.80		-11.13(.00)	-0.60	-5.47 (.00)	-0.25		-4.68 (.00)	-0.08	-0.89 (.74)
rLOC	-0.29		-2.04 (.00)	-0.42	-4.30 (.00)	-0.04		-0.61 (.46)	-0.11	-1.40 (.50)
lLOC	-0.36		-2.87 (.00)	-0.36	-4.66 (.00)	-0.12		-1.65 (.15)	0.00	-0.07 (.84)

While our main predictions concerned the MVPA results, we explored the possibility that our task led to particularly small univariate responses due to repetition suppression. Repetition suppression has been used as a tool to characterize functional specificity of neuronal populations, since neurons tend to adapt most to their preferred stimuli [54,55]. Previous studies have reported repetition suppression across runs for faces in face-selective areas [56–58].

The present task required displacement judgments (which are not highly attention-demanding) and a great deal of stimulus repetition (each image repeating 12 times per run). This combination likely fostered repetition suppression, and perhaps to a greater extent within category, for cars and faces, than for objects, since cars are visually (and semantically) more homogeneous. Thus, given both the stimulus characteristics and the fact that face-selective areas of individuals with above-average car recognition ability should be more selective for cars than for objects, we might expect more repetition suppression for cars and faces than objects, resulting in a paradoxically low average activation for these categories.

**Fig 5:**
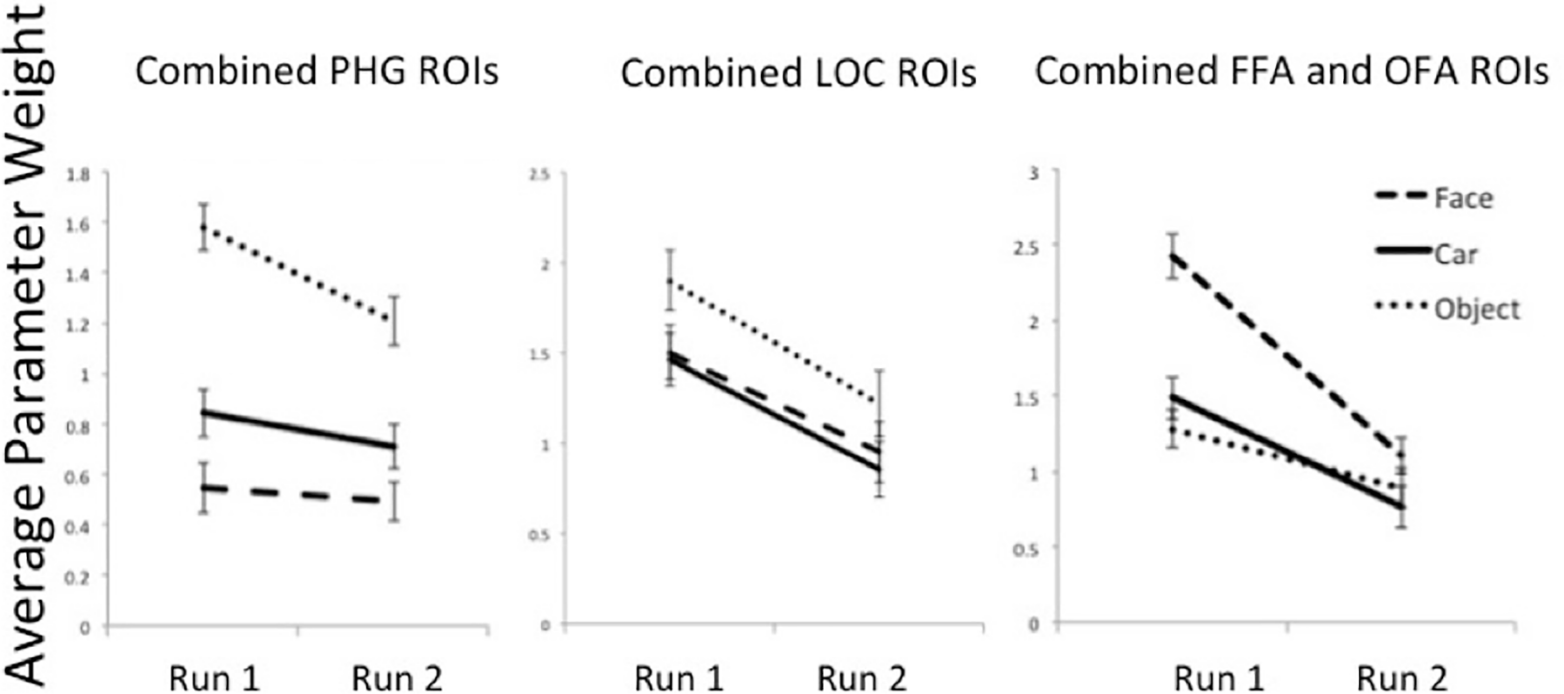
Average parameter weights for faces (dashed), objects (dotted) and cars (solid) plotted for perception runs 1 and 2 in combined face-selective regions (right, significant run*category interaction) and object-selective regions (left). Fixation is used as a baseline for the face, object and car conditions and error bars show SEM values.

We investigated this possibility by calculating parameter weights for each run using fixation as a baseline (see supplemental S1 Table). In general, faces and cars had higher parameter weights than objects in the first perception run, and faces and cars showing a larger decrease than objects between run 1 and 2 (see Fig 5). Of interest was the prediction of more habituation for faces and cars than for objects in face-selective areas specifically. This was supported by an ANOVA using factors of ROI type (face-selective ROIs, PHG ROIs and LOC ROIs), with perception runs (Run1 and Run 2 with weights of 1 and -1), and category (face, car and object with weights of 1, 1 and -2). This led to a significant interaction between ROI type, run and category (F(2,38) = 33.39, *p* < .0001, η_p_^2^ = .64). We unpacked this interaction with a run x condition ANOVA in each type of ROI. In face-selective ROIs, there was a run x category interaction (F(1,19 = 23.39, p = .0001, η_p_^2^ = .55), with more habituation for faces and cars than for objects. The same interaction was also significant in the PHG ROIs (F(1,19 = 9.75, p = .006, η_p_^2^ = .34), but in this case it reflected more habituation for objects than for faces and cars. The interaction was not significant in LOC ROIs (F(1,19 = 0.67, p = .42, η_p_^2^ = .03).

Because repetition suppression is an indication of functional specificity (e.g., [54]), these results are consistent with stronger selectivity of face-selective areas for faces and cars than for objects in our sample and they suggest that mean responses across runs in this design are not a good indication of selectivity.

## Discussion

We set out to determine if the FFA can support imagery of non-face objects at the subordinate-level. To answer this question, we compared how well classifiers could distinguish between imagined faces, cars and objects in a sample of above-average car recognizers. We found that a classifier trained and tested on imagery runs can distinguish between imagined cars and imagined objects in some face-selective regions, suggesting that car representations exist in face-selective areas during visual imagery.

In addition, a classifier trained on imagery runs can successfully distinguish perceived cars from perceived objects in the anterior FFA (FFA2), an area that has shown the most robust car expertise effects in prior work [31,34] and also seems particularly sensitive to experience for faces [32,33]. These across-task results further reveal that the representations of cars in our participants are at least in some way similar to those evoked during perception. Importantly however, our results refute the idea that face-selective areas are selectively recruited for face imagery [4]. Future work using an event-related design could further this finding by determining if similar results could be found when training and testing at the subordinate level.

In fact, the results generally suggest that faces and cars can be decoded from objects in several non-face selective areas. Such results are consistent with the general idea that categorical representations are distributed (e.g., [14]). The parahippocampal gyrus, for instance, tends to show a different response to faces than non-face objects (Martin et al. 2013), has been found be engaged for objects of expertise [25], and its response to faces relative to objects predicts face recognition ability [33].

Though our main analyses focused on distinguishing imagined cars from imagined objects when training and testing on imagery data, we should note that this within-task (imagery to imagery) classifier appeared to show somewhat weaker decoding of faces from objects than cars from objects. That is, imagined faces vs. imagined objects decoding was only above statistical significance in one face-selective region (lFFA2). We do not wish to over-interpret this relatively weak decoding for faces in this condition, because we do not have sufficient statistical power to show that decoding in lFFA2 is larger than that in other face-selective ROIs or that the decoding for cars was stronger than that for faces. Instead, we would emphasize that the cross-trained analyses (training on imagery and testing on perception) demonstrate that, in most face-selective areas, representations of imagined faces and objects are sufficiently similar to representations of their perceived counterparts to allow training of a classifier that can decode representations of perceived faces and objects. Perhaps what these results highlight is that there may be factors at play that we do not presently have a good way to measure. For instance, we know little about the relative difficulty of imagining objects from different categories, such as cars and faces, which could recruit different strategies, thereby facilitating decoding. While we can measure the neural correlates of imagery, there are few methodological approaches that allow us insight into the quality of the images our participants generate, aside from their similarity to perceptual representations.

Though we made no predictions about the face versus car classification, we note that previous work found a negative correlation between car recognition ability and face versus car classification performance [31]. Here, we find that decoding faces from cars is possible in some face-selective areas for both our within-task classifier (bilateral FFA1s and FFA2s) and our across-task classifier (lFFA1, lFFA2 and lOFA, see Figs 3 and 4). It is important to highlight that the present methods can reveal whether decoding is possible, but do not provide information as to the differences between representations, which are suggested by the well-below perfect decoding performance. Based on McGugin et al. [31], we might speculate that faces and cars would be more easily distinguished in car novices than in our current sample. It is certainly interesting that the rFFA2, which has emerged in other studies as the main area sensitive to expertise [28,31,33], can distinguish both perceived faces and perceived cars from perceived objects based on imagery training, but cannot distinguish faces vs. cars in the same across-task comparisons. This region may be where car and face representations are most similar in car experts, in imagery as in perception.

We did not systematically compare within- and across-task analyses, because the across-task analysis uses more data (all three imagery runs are used for training the classifier instead of two runs, and all the perception runs are used for decoding rather than only one imagery run). However, a qualitative comparison suggests that decoding is not always best when more data is used, so it is interesting to speculate on this qualitative comparison. In the extreme, if the representations used during perception and imagery were identical, then we would expect across-task classification to always be better than within-task classification because more data is available. This is generally the case for the contrast of faces vs. objects, where decoding was better in across- than within-task analyses in almost every ROI (see S1 Fig for a qualitative comparison). It was also the case for decoding faces vs. cars in left face-selective ROIs and cars vs. objects in right object-selective ROIs. In some cases (most clearly in decoding faces vs. cars in object-selective areas) the across- and within-task classifiers performed similarly.

However, another consideration when comparing the across- and within-task analyses is that the across-task analysis trains and decodes on different tasks, whereas the within-task analysis uses a leave-one-out approach in a homogeneous set of runs. In that sense, even though the within-task analysis uses less data, it may provide better decoding than the across-task analysis *to the extent that the representations used during imagery and perception differ*. It is interesting that within-task decoding appears to outperform across-task decoding mainly in the car vs. object comparison. This could mean that some of what our participants do during imagery may not overlap with perception (more for cars than faces and objects), and yet still be category specific (sufficient to distinguish cars from objects). For instance, despite being asked to imagine the specific images used during the perception task, each car expert may have his or her own preferred representation of the car models we used (e.g. instead of imagining our image of a Kia Forte, they imagine a different image of a Kia Forte). This is obviously speculative but it presents a challenge to the suggestion by Cichy et al [5] that all features of an imagined representation should overlap with those in perceived representations.

In our univariate analysis, we did not entirely replicate previous findings [4–6] of greater activation for faces in face-selective areas during visual imagery, as we only found this effect in lFFA2. This discrepancy could be due to several factors. First, unlike prior work we divided our face-selective regions into subregions (FFA1 and FFA2), rather than picking the more face-selective peak or averaging the peaks together. Second, the contrast we used in our univariate analysis compared activation during face and car imagery to object imagery activation. This contrast differs from previously used contrasts of faces versus places [4], faces versus non-faces (bodies, objects, scenes and houses, [5]) and faces versus houses and chairs [6]. Additionally, O’Craven and Kanwisher [4] reported that in some participants, they did not find overlap between perception and imagery responses, perhaps due to lower visual imagery ability. Because our sample was selected to be above average on the VVIQ, this seems unlikely to explain the present results. Finally, our participants were men recruited to have above-average car recognition ability, which could explain some of these differences, although their average CFMT performance was not different from that in larger unselected samples (*t*(122) = 1.05; *p* = .30, d = 0.23, larger unselected sample of 104 from [59]). Regardless of the reason, our study was designed for multivariate analysis and prior work has shown multivariate analyses can be informative despite weak or absent univariate effects (e.g., [60]). Additionally, given that all our participants were above-average in car recognition (Table 2), the fact that we did not find greater activation for perceived cars relative to perceived objects may seem odd given that many prior studies reported car expertise effects in the face-selective areas of car experts. However, prior work relied on correlations between car recognition ability and selectivity for cars in face-selective areas rather than absolute values, so it is difficult to compare across these designs (e.g., [25,27,28]). Importantly, in the present participants, who were car experts, cars showed habituation that was similar to faces both in face-selective areas (more habituation than for objects) and in the PHG (less habituation than for objects). Future work could compare repetition suppression for cars in car experts and novices to confirm our interpretation of these results as resulting from car expertise. While we did not vary car expertise in this study, we attribute decoding of cars from objects in FFA during imagery to perceptual expertise. However, we acknowledge that if car novices were tested while they imagined various cars, they could also recruit face-selective areas for this task.

Our results provide new evidence that visual imagery mirrors visual perception, not only in early visual areas [22], but also further down the visual processing stream. This also applies to a category for which detailed representations would have likely been acquired relatively late in life. Therefore, it appears that experience impacts not only how objects are represented in the brain during perception, but also during imagery. The nature of these representations, however, remains unclear. Though the work reported here and previous work shows that visual imagery can be characterized as a top-down re-instantiation of vision, it has yet to be determined whether these mental image representations are necessarily “visual” or if they draw more upon semantic representations associated with visual images.

## Supporting information

S1 Fig **Average accuracies of the across-task classifier (train imagery-test perception, Fig 4) overlaid on top of the within-task classifier (train imagery-test imagery, Fig 3) for object versus car (left) and face versus object (middle) and face versus car (right) two-way classifications in face-selective ROIs (upper row) and object-selective ROIs (lower row)**. Across-task classifier is in greyscale and translucent.
(DOCX)

S1 Table Run 1 and Run 2 parameter weights for the two perception runs. Fixation is used as the baseline. Run 2 parameter weights that are significantly lower than Run 1 parameter weights (one-tailed) are denoted with an asterisk.
(DOCX)

S2 Table Reports average classifier accuracies when the classifier is trained on imagery runs and then tested on perception runs and vice versa. In both Tables 2 and 3 we also report classification accuracies for an early visual cortex region (EVC). These regions (one right and one left) were a single set of 27 contiguous function voxels (729 structural voxels each; right EVC—mean X = 26.22 (SD = 2.94), mean Y = -88.22, (SD = 3.01), mean Z = -10.22 (SD = 2.98); left EVC—mean X = -24.67 (SD = 2.98), mean Y = -89.56 (SD = 2.83), mean Z = -9.00 (SD = 3.06)). The same set of voxels was used for each participant. All *t*-tests are one-tailed.
(DOCX)

S3 Table Reports average classifier accuracies when the classifier is trained on perception and then tested on imagery runs. (DOCX)
