# Supplementary material for: Visual imagery of faces and cars in face-selective visual areas
Source: PLoS One. 2018 Sep 28;13(9):e0205041. doi: 10.1371/journal.pone.0205041 (PMC6161903; doi:10.1371/journal.pone.0205041)

Fig S1. Average accuracies of the across-task classifier (train imagery-test perception, Fig 4) overlaid on top of the within-task classifier (train imagery-test imagery, Fig 3) for object versus car (left) and face versus object (middle) and face versus car (right) two-way classifications in face-selective ROIs (upper row) and object-selective ROIs (lower row). Across-task classifier is in greyscale and translucent.


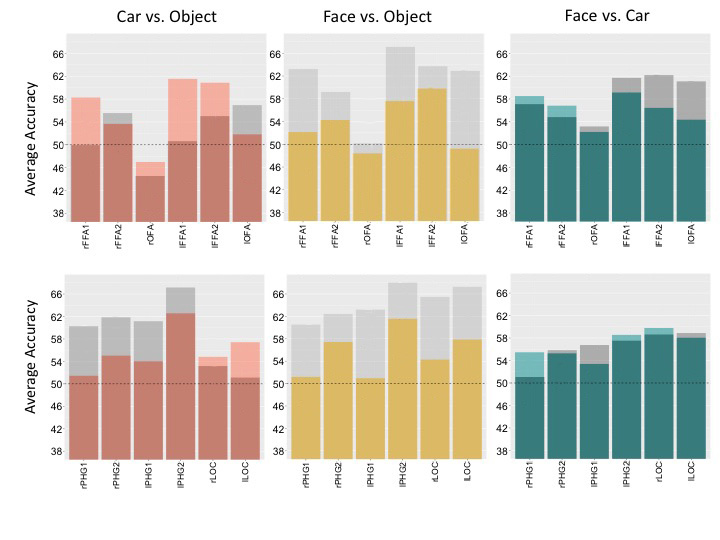

Supplement: S1 Fig — Average accuracies of the across-task classifier (train imagery-test perception, Fig 4) overlaid on top of the within-task classifier (train imagery-test imagery, Fig 3) for object versus car (left) and face versus object (middle) and face versus car (right) two-way classifications in face-selective ROIs (upper row) and object-selective ROIs (lower row). Across-task classifier is in greyscale and translucent. (DOCX) [file pone.0205041.s001.docx]
