# Supplementary material for: Visual imagery of faces and cars in face-selective visual areas
Source: PLoS One. 2018 Sep 28;13(9):e0205041. doi: 10.1371/journal.pone.0205041 (PMC6161903; doi:10.1371/journal.pone.0205041)
Supplement: S1 Table — Fixation is used as the baseline. Run 2 parameter weights that are significantly lower than Run 1 parameter weights (one-tailed) are denoted with an asterisk. (DOCX) [file pone.0205041.s002.docx]

Table S1. Run 1 and Run 2 parameter weights for the two perception runs. Fixation is used as the baseline. Run 2 parameter weights that are significantly lower than Run 1 parameter weights (one-tailed) are denoted with an asterisk.

|  | Face | | Object | | Car | |
| --- | --- | --- | --- | --- | --- | --- |
|  | Run 1 | Run 2 | Run 1 | Run 2 | Run 1 | Run 2 |
| rFFA1 | 3.25 | 1.67*** | 1.84 | 1.4* | 2.1 | 1.17*** |
| rFFA2 | 1.94 | 0.98** | 0.91 | 0.74 | 0.94 | 0.46* |
| rOFA | 2.46 | 1.2** | 1.39 | 1.26 | 1.84 | 1.18* |
| lFFA1 | 2.51 | 1.28*** | 1.51 | 0.76** | 1.38 | 0.94* |
| lFFA2 | 1.42 | 0.42*** | 0.41 | 0.16 | 0.56 | -0.15*** |
| lOFA | 3.59 | 1.44** | 2.18 | 1.4 | 2.72 | 1.54** |
| rPHG1 | 0.77 | 0.46 | 1.71 | 1.29* | 1.11 | 0.83 |
| rPHG2 | 0.17 | 0.37 | 1.39 | 1.01* | 0.44 | 0.49 |
| lPHG1 | 0.95 | 0.64* | 1.9 | 1.43** | 1.27 | 0.92 |
| lPHG2 | 0.3 | 0.5 | 1.32 | 1.1 | 0.56 | 0.61 |
| rLOC | 1.57 | 1.22** | 1.92 | 1.46** | 1.57 | 0.95** |
| lLOC | 1.44 | 0.68 | 1.89 | 0.97*** | 1.36 | 0.76** |

*p<.05, ** p<.01, *** p<.001
