# Supplementary material for: Visual imagery of faces and cars in face-selective visual areas
Source: PLoS One. 2018 Sep 28;13(9):e0205041. doi: 10.1371/journal.pone.0205041 (PMC6161903; doi:10.1371/journal.pone.0205041)
Supplement: S2 Table — In both Tables 2 and 3 we also report classification accuracies for an early visual cortex region (EVC). These regions (one right and one left) were a single set of 27 contiguous function voxels (729 structural voxels each; right EVC—mean X = 26.22 (SD = 2.94), mean Y = -88.22, (SD = 3.01), mean Z = -10.22 (SD = 2.98); left EVC—mean X = -24.67 (SD = 2.98), mean Y = -89.56 (SD = 2.83), mean Z = -9.00 (SD = 3.06)). The same set of voxels was used for each participant. All t-tests are one-tailed. (DOCX) [file pone.0205041.s003.docx]

Table S2. reports average classifier accuracies when the classifier is trained on imagery runs and then tested on perception runs and vice versa. In both tables 2 and 3 we also report classification accuracies for an early visual cortex region (EVC). These regions (one right and one left) were a single set of 27 contiguous function voxels (729 structural voxels each; right EVC - mean X = 26.22 (SD = 2.94), mean Y = -88.22, (SD = 3.01), mean Z = -10.22 (SD = 2.98); left EVC - mean X = -24.67 (SD = 2.98), mean Y = -89.56 (SD = 2.83), mean Z = -9.00 (SD = 3.06)). The same set of voxels was used for each participant. All *t*-tests are one-tailed.

|  | Train Imagery – Test Imagery | | | Train Imagery – Test Perception | | |
| --- | --- | --- | --- | --- | --- | --- |
|  | Face vs Car | Face vs Obj | Obj vs Car | Face vs Car | Face vs Obj | Obj vs Car |
| lFFA1 | 59.13* | 57.6 | 61.54*** | 61.71** | 67.19*** | 50.64 |
| lFFA2 | 56.44* | 59.79* | 60.86** | 62.18** | 63.71*** | 55.02* |
| lOFA | 54.37 | 49.23 | 51.81 | 61.08*** | 62.90** | 56.94 |
| rFFA1 | 58.51* | 52.16 | 58.27** | 57.09 | 63.22*** | 49.95 |
| rFFA2 | 56.83* | 54.27 | 53.65 | 54.82 | 59.19** | 55.56* |
| rOFA | 52.22 | 46.39 | 46.98 | 53.18 | 50.17 | 44.55 |
| lPHG1 | 53.38 | 50.94 | 54.01 | 56.76* | 63.2 | 61.16** |
| lPHG2 | 58.55* | 61.57** | 62.56*** | 57.53* | 68.02*** | 67.17*** |
| rPHG1 | 55.46* | 51.18 | 51.43 | 51.08 | 60.52*** | 60.27*** |
| rPHG2 | 55.27* | 57.41* | 55.02* | 55.84 | 62.44** | 61.86*** |
| lLOC | 58.05* | 57.87* | 57.41** | 51.14** | 67.27** | 51.14 |
| rLOC | 59.79*** | 54.28 | 54.80 | 53.18* | 65.47*** | 53.18 |
| lEVC | 48.88 | 50.06 | 49.04 | 54.8 | 55.42 | 57.56* |
| rEVC | 50.42 | 50.44 | 49.63 | 51.1 | 52.24 | 48.88 |

*p<.05, ** p<.01, *** p<.001

Note: All averages survive FDR correction.
