# Supplementary material for: Visual imagery of faces and cars in face-selective visual areas
Source: PLoS One. 2018 Sep 28;13(9):e0205041. doi: 10.1371/journal.pone.0205041 (PMC6161903; doi:10.1371/journal.pone.0205041)
Supplement: S3 Table — (DOCX) [file pone.0205041.s004.docx]

Table S3. reports average classifier accuracies when the classifier is trained on perception and then tested on imagery runs.

|  | Train Perception – Test Imagery | | |
| --- | --- | --- | --- |
|  | Face vs Car | Face vs Obj | Obj vs Car |
| lFFA1 | 58.84 | **58.70*** | 47.44 |
| lFFA2 | **59.60**** | **58.06*** | 53.80 |
| lOFA | **57.27**** | 50.64 | 50.71 |
| rFFA1 | 56.32 | **58.95**** | 51.36 |
| rFFA2 | 55.38* | **55.68*** | 54.42 |
| rOFA | 50.43 | 50.68 | 49.75 |
| lPHG1 | 56.94* | **56.73*** | **55.60**** |
| lPHG2 | 53.30 | **61.33**** | **63.32***** |
| rPHG1 | 49.91 | **55.86*** | **55.88**** |
| rPHG2 | 51.36 | **56.90**** | **56.16**** |
| lEVC | 53.99 | 53.72 | 48.65 |
| rEVC | 52.94 | 52.31 | 51.01 |

*p<.05, ** p<.01, *** p<.001

Note: Bolded averages survive FDR correction.
